# Low-dose intravenous esketamine on a depressive catatonia patient with venous thromboembolism: a case report

**DOI:** 10.3389/fpsyt.2025.1688633

**Published:** 2025-11-06

**Authors:** Meng-han Zhang, Yi-fan Wang, Juan Li, Zi-jun Liu, Xiao Ji

**Affiliations:** 1 Beijing Key Laboratory for the Diagnosis and Treatment of Mental Disorders, National Clinical Research Center for Mental Disorders, Beijing Anding Hospital, Capital Medical University, Beijing, China; 2 High Precision Innovation Center for Human Brain Protection, Capital Medical University, Beijing, China

**Keywords:** catatonia, major depressive disorder, esketamine, N-methyl-D-aspartate receptor antagonist, venousthromboembolism

## Abstract

**Background:**

Catatonia is a rare but potentially life-threatening psycho-motor syndrome. It is mainly manifested as decreased activities, or excessive and specific activities, such as stupor, agitation and defiance. Catatonia can appear in various diseases and the diagnosis and treatment of depressive catatonia are often delayed. As an N-methyl-D-aspartate (NMDA) receptor antagonist, esketamine shows antidepressant and anti-catatonic effects by regulating glutamatergic signal transduction and enhancing synaptic plasticity.

**Methods:**

The present case involved a 55-year-old woman with depressive catatonia. Benzodiazepines, aripiprazole and nutritional support were given after admission. As she had a poor response to the initial treatment, we considered using the modified electroconvulsive therapy (MECT). Due to the deep vein thrombosis in the lower extremities, the application of MECT was restricted. With the consent of her and family, we decied to administrate a sub-anesthetic dose of intravenous (IV) Esketamine (0.2 mg/Kg) combined with 50mg desvenlafaxine. During the process, we assessed the effect by comparing depression severity scores using validated psychiatric scales before and after the treatment. Adverse drug reactions were evaluated by adverse drug reaction scale.

**Result:**

The symptoms of catatonia were relieved in 4 hours after esketamine administration. The Bush-Francis Catatonia Rating Scale (BFCRS) decreased from 19 to 0. 48 hours after injection, the depressive symptoms were relieved and the Montgomery Åsberg Depression Rating Scale (MADRS) decreased from 46 to 9. The condition remained stable on 20 weeks of follow-up.

**Conclusions:**

As a safe and rapid intervention, Esketamine might be a new option for catatonic patients who cannot undergo MECT or fail to respond to conventional treatment. It may be worthy of further research in the future.

## 1 Introduction

Catatonia is a severe neuropsychiatric syndrome that affects mood, speech, movement, and complex behavior (1). It can occur in a variety of psychiatric and neurological disorders, including depression, mania, schizophrenia, autism, autoimmune encephalitis, systemic lupus erythematosus, thyroid disorders, epilepsy, drug-induced and drug withdrawal (1). Catatonia can appear in a variety of disease contexts, among which, the diagnosis and treatment of depressive catatonia are often delayed. As catatonia may lead to life-threatening consequences such as pneumonia, pressure ulcers, sepsis, deep vein thrombosis, and pulmonary embolism (2), early identification and treatment are particularly important. Benzodiazepines and modified electroconvulsive therapy (MECT) are the main treatments for catatonia.

However, 20%-40% of catatonic patients do not improve with MECT, benzodiazepines or combination of both (3–6). Excessive activation of glutamate and N-methyl-D-aspartate (NMDA) receptors may be involved in the occurrence of catatonia (7). As an NMDA receptor antagonist, esketamine exerts its pharmacological effects by regulating glutamatergic signaling and enhancing synaptic plasticity. An option may be offered to catatonic patients who do not respond well to benzodiazepines or MECT. This case report described a successful use of IV esketamine in depressive catatonia with narrowed treatment options due to venous thromboembolism.

## 2 Case report

### 2.1 Chief complaints

The patient was a 55-year-old woman with major depressive disorder, which manifested as depressive catatonia with stupor, mutism, and refusal to eat.

### 2.2 History of present illness

The family described a 1-year history of worsening mood symptoms, characterized by a depressive syndrome, decreased activity, decreased appetite, and abnormal sleep. One month before admission, the condition worsened, accompanied by anxiety, dizziness, and vomiting. She remained bedridden all day long, refusing to socialize. Due to the symptoms of dizziness and vomiting, she had visited the neurology department several times and was diagnosed with “cerebrovascular disease, gastroenteritis, hyponatremia and hypochloremia” before she visited the psychiatry department,. After nutritional support treatment, dizziness and vomiting were relieved in a short period of time. Three days before visiting the psychiatry department, she suddenly became excited, talkative, with disordered speech content and increased activity. In the morning of the next day, she went silent, inactive and did not actively urinate or defecate. From then on, she remained bedridden.

### 2.3 Past medical history

Past medical history included hypertension. Surgical history included a hysterectomy for uterine fibroids. In addition, a pancreaticoduodenectomy was performed for the presence of a congenital cyst in the pancreatic duct.

### 2.4 Physical and mental examination

The body temperature was 37.6°C, blood pressure was 123/85mmHg, and heart rate was 125 beats per minute. She lay on bed with limbs in a fixed position and hypertonic muscles in extremities. On the day of admission, she was unable to cooperate with the mental examination, kept mute with little expression change, disobedient, and refused to eat.

### 2.5 Laboratory examinations

According to the diagnosis and treatment of catatonia, we performed cytological examination of cerebrospinal fluid, cerebrospinal fluid composition, virological and bacteriological detection, antibody examination of autoimmune encephalitis, and brain imaging. We found no diagnostically abnormal findings. The blood test showed that D-dimer was significantly elevated (7.72mg/L, normal range: 0.00-0.55 mg/L), and the lower extremity venous ultrasound showed multiple thrombosis in the lower extremity veins. With her blood C-reactive protein level was elevated (1.01mg/dL, normal range: 0.00-0.80mg/L) and the chest CT report showed patchy high-density shadows in the right lower lobe, pneumonia was considered.

### 2.6 Diagnostic assessment and treatment

The patient was admitted medically for rehydration and nasogastric (NG) feeding. Anti-infective therapy with ceftriaxone was administered. In addition, rivaroxaban was used for the treatment of venous thromboembolism of the lower extremities.

Because the patient needed to clarify the etiological diagnosis of catatonia, we initially gave only lorazepam 0.5mg TID by nasal feeding or temporary IV diazepam 10mg for symptomatic treatment of catatonia. The patient’s tension symptoms were slightly improved, but the effect could not be maintained.The symptoms were more obvious in the morning. To improve the efficacy of the treatment, aripiprazole 5mg once daily was added (8). The baseline Bush-Francis Catatonia Rating Scale (BFCRS) was 27 points.

There was partial improvement in the patient’s psychiatric status after the initial of treatment. The mental examination showed that the consciousness was clear and her orientation was intact. She could only communicate in phrases. Her speech speed was slow, the voice was low, and her articulation was not clear. She reported low mood, feelings of hopelessness, decreased appetite, and decreased activity. Every morning she would become stuporous, stiff and silent. In the evening, she would have anxiety, explaining the sudden excitement in her medical history that she was worried and thought she was going to die. She had no hypomania, manic syndrome, or psychotic symptoms since the onset of the illness. According to the DSM-5 diagnostic criteria, the diagnosis was major depressive episode with catatonia.

Because the patient’s catatonia had only partially improved after the initial treatment, we considered administering MECT. However, she had a lower extremity venous thromboembolism, deeming her medically high-risk for MECT. After understanding the treatment risks, the patient and the family refused to MECT. As an alternative treatment for MECT for her severe depression, trial of IV esketamine was commenced. We considered the use of esketamine treatment, which has been reported to be effective in patients with catatonia (9, 10). Prior to commencement, Montgomery Åsberg Depression Rating Scale (MADRS) was 46 and BFCRS was 19. The esketamine was administered IV over 40 min with the dose of 0.2 mg/kg(totally 9.6mg) once. During the administration, an anesthesiologist was present during the process and post-treatment adverse reaction was monitored. A vital signs monitor was used to screen the patient’s electrocardiogram (ECG), blood pressure, and blood oxygen saturation. After infusion, drug adverse reaction scales and clinical dissociation symptom scales were used to assess the safety of esketamine.

### 2.7 Clinical outcomes and follow-up

Within 4 hours of the administration, the patient returned to fluent verbal communication,and catatonia was resolved with expressed improved emotions. She did not report any dissociation symptoms or discomfort. The monitor showed no abnormal changes in blood pressure or ECG. The BFCRS decreased from 19 to 0, and the MADRS decreased from 46 to 9.

Subsequently, the patient was treated with desvenlafaxine 50 mg per day for antidepressant. Considering the simplification of the remedy, we discontinued the use of aripiprazole. In the second week of the treatment, the dosage of lorazepam was reduced as well. After one week of anti-infection treatment for the pneumonia, the infection was getting better and the antibiotic treatment was discontinued. After the discharge, she took the medication regularly and was followed up regularly during 20-week period. She returned to her pre-illness state and enjoyed her life.

**Figure 1:**
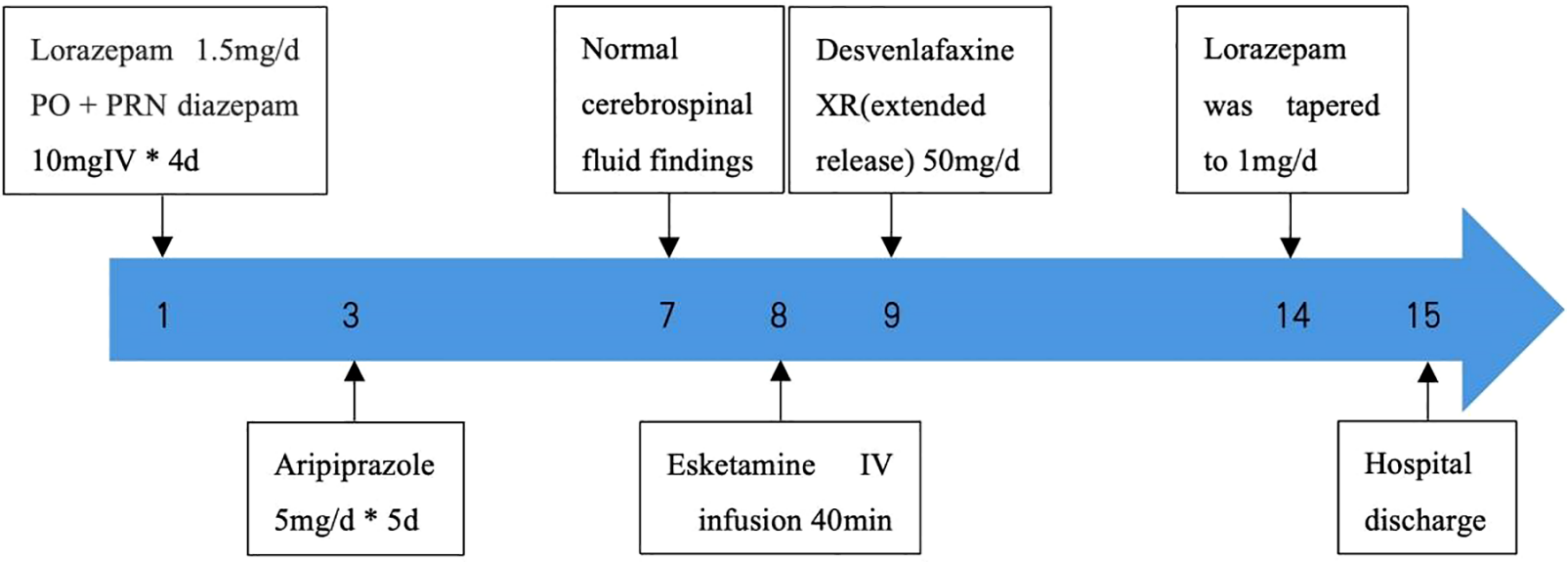
The treatment course.

**Figure 2:**
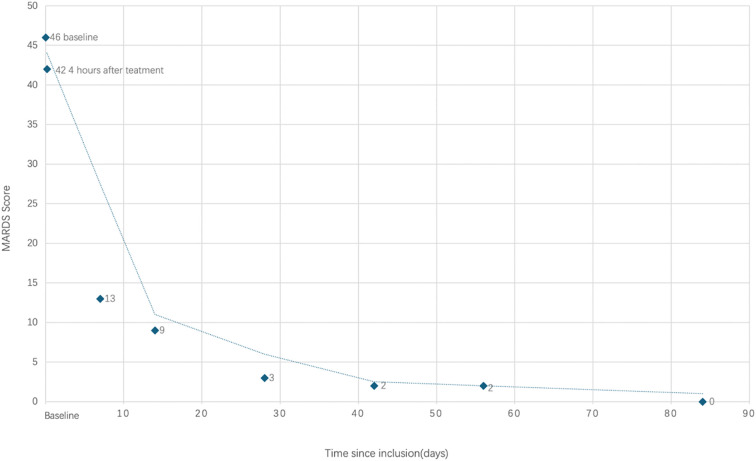
The changes in scores on the MADRS.

**Figure 1** shows the full treatment course. **Figure 2** shows the changes in scores on the MADRS.

## 3 Discussion

Catatonia is a rare but potentially life-threatening psychomotor syndrome with a global incidence of 10/100,000 person-years (1, 11, 12). Among the psychiatric disorders associated with catatonia, catatonia was thought to be mainly related to schizophrenia in the early years, but it has been found to be more common in affective disorders in recent years (8, 13). The pathophysiology of catatonia is still unclear. Patients with catatonia have been found to have abnormal activities of glutamate, dopamine and gamma-Aminobutyric Acid (GABA) (14). Overactivity of glutamate and over-activation of NMDA receptors may contribute to catatonia (14). It has been hypothesized that in catatonia, NMDA receptors in striatal-cortical or cortical-cortical pathways may be dysfunctional (15). In catatonia, allosteric effects of NMDA receptors on glutamate may lead to dysfunction of the cortical-striatal-thalamocortical (CSTC) circuit (11). NMDA receptor antagonists may reset the problems associated with dopamine and GABA depletion in the CSTC circuit by balancing the effects of NMDA receptors on GABA-A-parvalbumin interneurons in the prefrontal cortex (PFC). GABA-parvalbumin interneurons inhibit PFC pyramidal corticostriatal glutamatergic projections to the striatum. It also reduces NMDA action in the striatum itself (9). Previous case reports and systematic reviews have mainly discussed amantadine and memantine as major NMDA receptor antagonists for the treatment of catatonia (9).

Ketamine is an NMDA receptor antagonist composed of an equal mixture of (R)- and (S) -enantiomers, in which the (S) -enantiomer, esketamine, has a significantly higher affinity for NMDA receptors than the (R) -enantiomer (16). The mechanism of esketamine in the treatment of catatonia has not been elucidated. There were only 2 cases were reported in the existing literature that ketamine and its derivatives improve depression-related catatonia. J. Romay’s team (17) improved catatonia by intranasal esketamine. In contrast, the team of A. Laurin (18) treated an elderly patient with depression-related catatonia with intravenous ketamine.

This case reported the use of intravenous low-dose esketamine for the treatment of depressive catatonia, who is a depressive patient with catatonia symptoms (mutism, rigidity, defiance, and agitation) as the main clinical manifestations. No adverse drug reactions such as dissociation occurred. In this patient, esketamine was administered with an initial resolution of catatonia and an improvement in depressive symptoms 4 hours after the administration of the drug. The reason for the late use of esketamine is that catatonic disorder due to another medical condition needed to be excluded at the early stage, and there is no clear recommendation in domestic and foreign guidelines or consensus for the use of esketamine in catatonic patients.

As described above in the review, ketamine or esketamine may have a role in the medical management of catatonia either as monotherapy or as an adjunct to existing therapies, especially when these treatments are ineffective or contraindicated. In the future, more rigorous randomized controlled trials are needed to verify the efficacy and safety.

## Data Availability

The raw data supporting the conclusions of this article will be made available by the authors, without undue reservation.
